# *Rickettsia aeschlimannii* in Spain: Molecular Evidence in *Hyalomma marginatum* and Five Other Tick Species that Feed on Humans

**DOI:** 10.3201/eid0907.030077

**Published:** 2003-07

**Keywords:** *Rickettsia aeschlimannii*, Mediterranean spotted fever, PCR, tick, *Hyalomma*, *Haemaphysalis*, *Ixodes*, *Rhipicephalus*, letter

**To the Editor:** *Rickettsia aeschlimannii* is a pathogenic spotted fever group rickettsia first isolated from *Hyalomma marginatum* ticks collected in Morocco in 1997 (*1*). Later found in *H. marginatum* ticks from Zimbabwe, Niger, Mali, and Portugal (*2*), *R.* *aeschlimannii* has also been found in a *Rhipicephalus appendiculatus* tick attached to the right thigh of a patient in South Africa (*3*). These data suggest a broad geographic distribution for *R. aeschlimannii* and the possibility that tick species other than *H. marginatum* may also be suitable vectors for this rickettsia.

The pathogenicity of *Rickettsia aeschlimannii* in humans has been demonstrated by Raoult et al. (*4*) in a French patient who became ill after returning from a trip to Morocco. The patient exhibited symptoms similar to those of Mediterranean spotted fever (MSF) produced by *R. conorii*, with a tache noire–like eschar on his ankle, fever (39.5°C), and a generalized maculopapular skin rash. The second documented and most recent case of human infection by *R. aeschlimannii* occurred in a South African man who was bitten by *R. appendiculatus*; an eschar also developed around the tick attachment site on this patient (*3*). He was aware of the risk for tick-transmitted disease, so he removed the tick and administered doxycycline; he did not develop additional symptoms.

Over the past 6 years, throughout the region of Castilla y León, northwestern Spain, we have collected and identified 3,059 ticks belonging to 15 species (unpub. data) that were attached to persons living in this territory. We have systematically analyzed the ticks by polymerase chain reaction (PCR) to detect those infected with *Borrelia burgdorferi*, *Anaplasma phagocytophila*, and *Rickettsia* spp. This procedure enabled us to identify, for the first time in Spain, *R. aeschlimannii* in 35 tick specimens belonging to *H. marginatum* and to another five species.

During the 6-year study, ticks found on patients who sought medical advice in the hospitals and healthcare centers of Castilla y León were removed and referred to our laboratory for identification and analysis. Each tick was first disinfected by immersion in 70% alcohol, rinsed in sterile water, and dried on sterile filter paper. We then extracted DNA in 5% Chelex-100, according to the method of Guttman et al. (*5*). In searching for *Rickettsia* spp., we proceeded as described by La Scola and Raoult (*6*): All DNA samples were first tested for a fragment of the rickettsial *glt*A gene (*7*), and then, in the *glt*A-positive samples, a fragment of the rickettsial *omp*A gene (*8*) was amplified, sequenced, and compared with gene databases for identification. The *glt*A amplicon was sequenced only when the *omp*A was not successfully amplified. To prevent DNA contamination and the carryover of amplified products, we used sterile tools at all times and carried out each step of the analysis (extracting DNA, preparing the reaction mixture, and amplifying and analyzing the PCR product) in separated work areas. Two negative controls (Milli-Q water and DNA from laboratory-reared noninfected ticks) were included in each amplification trial. These controls were never amplified.

We obtained 21 *omp*A amplicons (629–632 bp) from 21 ticks. One amplicon, from a *Haemaphysalis punctata* tick, had 100% sequence identity with the *omp*A of *R. aeschlimannii* (GenBank accession no. U43800). The nucleotide sequences of the remaining 20 *omp*A amplicons shared >99% similarity with the *omp*A of *R. aeschlimannii*. These 20 amplicons were obtained from nine *Hyalomma marginatum*, five *Rhipicephalus bursa*, three *R. turanicus*, one *R. sanguineus*, and two *Ixodes ricinus* ticks.

In an additional 14 ticks (10 *H. marginatum*, 2 *R. bursa*, 1 *R. sanguineus,* and 1 *I. ricinus*) we sequenced 14 *glt*A amplicons (382 bp), which were 100% identical to the *glt*A of *R. aeschlimannii* (GenBank accession no. U59722). No other tick-borne pathogens were detected in the 35 *R. aeschlimannii*–containing ticks.

*R. aeschlimannii* has never been detected in Spain; therefore, our study constitutes its first citation in this country. Because *R. aeschlimannii* had been already detected in ticks in Portugal, we believe that its presence in Spain was expected and our finding is not surprising. However, the high number of tick species in which we found this rickettsia was unexpected: six species belonging to four genera. For these six species, the ratio between the specimens infected and the specimens analyzed (as well as the infection rates) were as follows: *I. ricinus* (3/1,320; 0.23%), *H. marginatum* (19/324; 5.86%), *H. punctata* (1/106; 0.94%), *R. bursa* (7/425, 1.64%), *R. sanguineus* (2/102; 1.96%), and *R. turanicus* (3/330; 0.91%). Although *H. marginatum* was the fourth most anthropophilic species in our study, this species simultaneously showed the highest number of infected specimens and the highest infection rate, making *H. marginatum* the main vector of *R. aeschlimannii* in our region. The next most important vectors are *Rhipicephalus* spp., and in particular *R. bursa*.

The 35 *R. aeschlimannii*–positive ticks were removed in the first 6 to 12 hours after attachment, before they could have ingested any blood, thus indicating that they were previously infected with the bacterium. Persons bitten by these specimens had never had symptoms of spotted fever, and they remained asymptomatic after the bite, suggesting that, as expected because of the rapid removal of the ticks, they did not acquire the infection.

Although MSF is endemic in Castilla y León (*9*), we only found one tick infected with *R. conorii* (0.03%) among the 3,059 analyzed (unpub. data), whereas *R. aeschlimannii* was much more prevalent in these same ticks (1.14%). Hence, in accordance with what was proposed by Raoult et al. (*4*) for MSF cases in Morocco, we suspect that many cases of MSF in Castilla y León may really have been due to *R. aeschlimannii*.

Our findings show that *R. aeschlimannii* is present in Castilla y León, the largest region in Spain, in six tick species that frequently feed on humans. Our observations expand the geographic distribution of this bacterium and the range of its potential tick vectors.
